# RET signaling pathway and RET inhibitors in human cancer

**DOI:** 10.3389/fonc.2022.932353

**Published:** 2022-07-25

**Authors:** Angelina T. Regua, Mariana Najjar, Hui-Wen Lo

**Affiliations:** ^1^ Department of Cancer Biology, Wake Forest School of Medicine, Winston-Salem, NC, United States; ^2^ Wake Forest Baptist Comprehensive Cancer Center, Winston-Salem, NC, United States

**Keywords:** RET, cancer, therapeutics, lung cancer, thyroid cancer

## Abstract

Rearranged during transfection (RET) receptor tyrosine kinase was first identified over thirty years ago as a novel transforming gene. Since its discovery and subsequent pathway characterization, RET alterations have been identified in numerous cancer types and are most prevalent in thyroid carcinomas and non-small cell lung cancer (NSCLC). In other tumor types such as breast cancer and salivary gland carcinomas, RET alterations can be found at lower frequencies. Aberrant RET activity is associated with poor prognosis of thyroid and lung carcinoma patients, and is strongly correlated with increased risk of distant metastases. RET aberrations encompass a variety of genomic or proteomic alterations, most of which confer constitutive activation of RET. Activating RET alterations, such as point mutations or gene fusions, enhance activity of signaling pathways downstream of RET, namely PI3K/AKT, RAS/RAF, MAPK, and PLCγ pathways, to promote cell proliferation, growth, and survival. Given the important role that mutant RET plays in metastatic cancers, significant efforts have been made in developing inhibitors against RET kinase activity. These efforts have led to FDA approval of Selpercatinib and Pralsetinib for NSCLC, as well as, additional selective RET inhibitors in preclinical and clinical testing. This review covers the current biological understanding of RET signaling, the impact of RET hyperactivity on tumor progression in multiple tumor types, and RET inhibitors with promising preclinical and clinical efficacy.

## Introduction

Since the discovery of the first tyrosine kinase in the early 1980s, identification of aberrant tyrosine kinase activity in cancers have made them optimal targets for therapeutic intervention in malignant diseases. Rearranged during transfection (RET) is one such receptor tyrosine kinase first discovered in 1985 as a novel transforming gene in NIH3T3 cells upon transfection with DNA isolated from human lymphoma cells (1), resulting in their oncogenic transformation. Using Southern blot analysis, Takahashi and colleagues determined that the putative transforming gene was a product of two separate DNA sequences that were >25kb apart on the human genome, and therefore was the result of genetic recombination that produced a transcriptionally functional unit. This novel transforming gene was subsequently named for the process through which it was discovered.

Following its discovery, RET has been identified to play critical roles in numerous developmental processes, namely embryonic development of the kidney (2, 3), as well as, the enteric nervous system (2, 4). Accordingly, RET alterations are implicated in numerous disease phenotypes such as Hirschsprung’s disease and cancer (5, 6). Additionally, studies performed over the last three decades have identified numerous alterations in RET that confer constitutive activation of its kinase activity, which is considered a causative factor in many cancer subtypes (7–10). Given that aberrant RET activity is an oncogenic driver in numerous cancers, RET is considered an optimal target for therapeutic intervention, first with multi-kinase inhibitors which were soon followed by the first generation of selective RET inhibitors. This review will summarize the current understanding of canonical RET signaling and its biology, the role of RET signaling in critical developmental processes, and the oncogenic role of aberrant RET activity in multiple cancer types. This review will also discuss the alterations in RET that result in its constitutive and oncogenic activation, the suboptimal clinical benefits of repurposed multi-kinase inhibitors for RET inhibition, and the compounds developed to selectively target RET kinase activity.

## Canonical RET signaling

**Figure 1:**
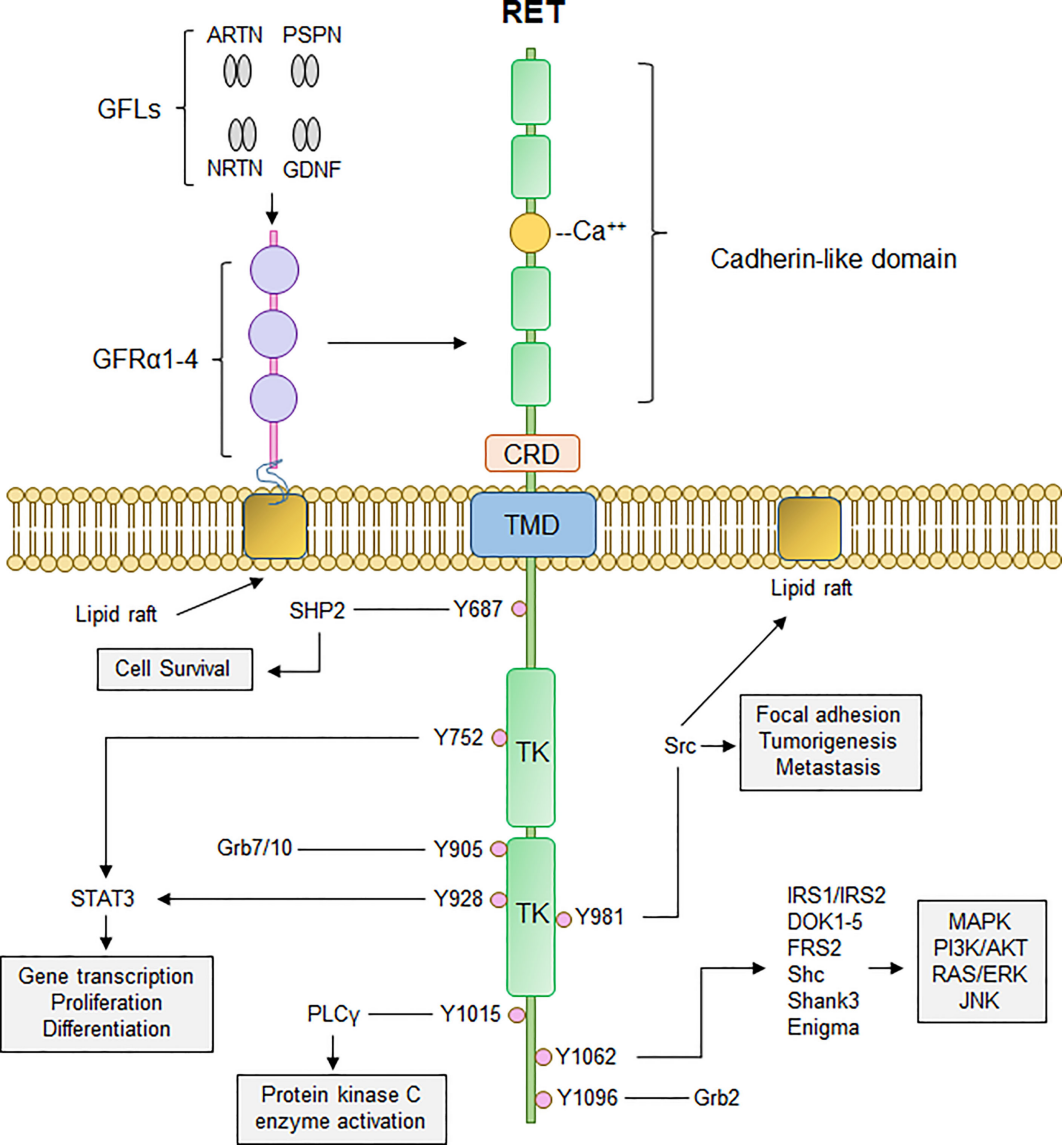
Canonical RET signaling. RET activation occurs upon fulfillment of multiple steps. Binding of GDNF-family ligands (GFLs) such as Artemin, Neurturin, Persephin, (ARTN, NRTN, PSPN, respectively), and GDNF to co-receptor GFRα1-4, concurrently with binding of calcium ions to the calcium binding domain, induces recruitment of RET, forming RET-GFRα complex. Formation of RET-GFRα complex brings two RET monomers in close proximity to induce homodimerization and crossphosphorylation of key RET tyrosine residues that recruit adaptor proteins important for propagation of RET signaling, such as PI3K/AKT, MAPK, PLCγ, and RAS/RAF/ERK. Thus, activation of RET signaling ultimately promotes cell proliferation, growth, and survival through activation of multiple downstream signaling cascades. CRD, cysteine-rich domain; TMD, transmembrane domain; TK, tyrosine kinase domain.

RET is a transmembrane receptor tyrosine kinase with a unique extracellular domain that contains four cadherin-like domains and 16 cysteine residues within a 120 amino acid sequence (11). RET activation occurs upon fulfillment of multiple events: binding of Ca^2+^ ions to its cadherin-like domains, binding of GDNF family ligands (GFLs; GDNF, ARTN, NRTN, PSPN) to glycosyl phosphatidylinositol-anchored co-receptor GDNF family receptor-α (GFRα1-4), resulting in recruitment of two RET receptors into close proximity and allowing for dimerization and autophosphorylation of several tyrosine residues on the cytoplasmic tails (12). Phosphorylation of these cytoplasmic tyrosine residues of RET allows for recruitment and binding of several adaptor proteins necessary for signal propagation of external stimuli and subsequent activation of downstream signaling cascades, namely PI3K/AKT, RAS/RAF/MEK/ERK, JAK2/STAT3, and PLCγ pathways (**Figure 1**). RET-Y687 recruits and binds SHP2 phosphatase, allowing for activation of PI3K/AKT which, in turn, promotes cell survival (13). RET-Y752 and Y928 are critical docking sites for Signal Transducer and Activator of Transcription 3 (STAT3), allowing for STAT3 phosphorylation, activation, and nuclear translocation critical for transcription of STAT3 target genes (14). Phosphorylation of RET-Y905 stabilizes the active conformation of RET and is critical for binding of adaptor proteins Grb7/10 (9, 15), whereas RET-Y981 is critical for binding and activation of Src kinase (16). Phospholipase C-γ binds to phospho-RET (Y1015) which, in turn, activates protein kinase C (PKC) pathway (17). RET-Y1062 phosphorylation recruits a number of adaptor proteins imperative for activation of PI3K/AKT, RAS/RAF/MEK/ERK, and MAPK pathways (18). Finally, Grb2 binds to phospho-RET (Y1096) and acts a secondary interaction through which RET can activate RAS/RAF/MEK/ERK pathway and promote cell proliferation and differentiation (19, 20).

## Roles of RET in development of the embryonic kidney, autonomic and enteric nervous systems, and spermatogenesis

In non-tumor tissues, RET signaling is critical for normal embryonic kidney development. Pachnis et al. demonstrated that *c-ret* is expressed in migrating neural crest cells within the cranial region of E8.5-9.5 mouse embryos, as well as in the cranial ganglia (E10.5) in the autonomic ganglia within the embryonic trunk region of the peripheral nervous system, and the myenteric ganglia of the gut at E13.5 (21). RET expression is also found in the developing Wolffian duct (WD), whereas the GDNF ligand is expressed in the metanephric mesenchyme (MM) adjacent to the WD. RET-expressing cells in the ureteric bud (UB) within the WD respond to GDNF abundance within the MM, promotes invagination of the UB into the MM where it can initiate branching to develop the embryonic kidney (3). Ivanchuk et al. found that *RET* mRNA is highly expressed in 14-week-old human embryos, however *RET* expression appears to decrease with gestational age and into adulthood, suggesting that RET is only required for the embryonic development of human kidneys (22). Indeed, Schuchardt et al. determined that, while mice lacking *c-ret* expression survived until birth, they expire ~24 hours after birth, likely due to severe renal agenesis or dysgenesis, or defective digestive tract function (discussed below) (2). These findings were corroborated by reports by Enomoto et al. (23) and Jain et al. (5) in which loss of the co-receptor GFRα1 or expression of a dominant-negative mutant RET also conferred deficits in embryonic renal development, resulting in renal agenesis and postnatal lethality. Interestingly, *c-ret* expression is temporally and spatially controlled: the adult salivary gland and adult brain expressed the highest levels of *c-ret*, but with lower *c-ret* expression in the heart and spleen, and was largely absent in the adult kidney, liver, lung, ovaries, or testes (21). Thus, RET expression is temporally and spatially regulated within embryonic tissues, suggesting that RET contributes to specific stages in embryonic development.

In addition to its role in embryonic kidney development, RET expression is also imperative for development, maturation, and maintenance of the autonomic and enteric nervous systems (ENS), which underlies proper gastrointestinal (GI) function. Early studies by Enomoto et al. found that RET expression is essential for development and maturation of parasympathetic arm of the murine autonomous nervous system (24), as well as axonal growth and migration of sympathetic neurons (4). Given that mice lacking *c-ret* expression were found to have reduced numbers, or complete absence, of enteric neurons throughout the digestive tract that can result in neonatal lethality (2), RET expression and activity appears to be critical for embryonic development of the GI tract. While RET expression is abundant in the developing murine intestinal epithelium, RET expression is maintained in a subset of enteroendocrine and enterochromaffin cells in adult tissues (25), which suggests a need for RET signaling in GI tract motility and/or secretion throughout adulthood. The GI phenotypes associated with loss of RET expression or activity are now characteristic of Hirschsprung’s disease (HD or HSCR), a congenital condition in which enteric nerves are missing in portions of the digestive tract (26). Since RET expression is critical for migration of neural crest cells and enteric neurons into the GI tract, it is unsurprising that loss of murine *c-ret* expression or activity induces GI phenotypes associated with HD, such as intestinal aganglionosis, and is detected in 50% of familial HD and up to 20% of spontaneous HD cases (6).

Several studies also implicated GDNF-RET signaling in spermatogenesis and determination of spermatogonial stem cell (SSC) fate. Spermatogonial maturation is dependent on renewal of SSCs. GDNF ligand is secreted by Sertoli cells (27), is essential for maintenance of SSCs *in vitro* (28), and SSC cell fate maturation *in vivo* (29). In addition to defective GI and renal development, Jain and colleagues reported defective spermatogenesis in mice expressing dominant-negative human RET (5). These findings were substantiated by Naughton et al. (30), in which they demonstrated that RET and GFRα1 are co-expressed in germ cells and SSCs during early spermatogenesis, and that the GDNF-RET-GFRα1 signaling axis is required for complete spermatogenesis and SSC self-renewal in a testis-autonomous transplantation mouse model, though this does not affect Sertoli cells (30).

## Aberrant RET signaling in tumorigenesis

The proto-oncogenic role of RET has been identified and characterized in numerous cancer types, such as, thyroid, lung, and breast cancers. RET alterations occur in less than 5% of all cancer patients (31, 32), however activating RET alterations, such as genetic amplification and chromosomal rearrangements, are found in increasingly higher proportions of specific cancer types. This section of the review will discuss oncogenic RET alterations and their implications in RET-altered cancers.

### Oncogenic RET alterations and RET fusions

**Figure 2:**
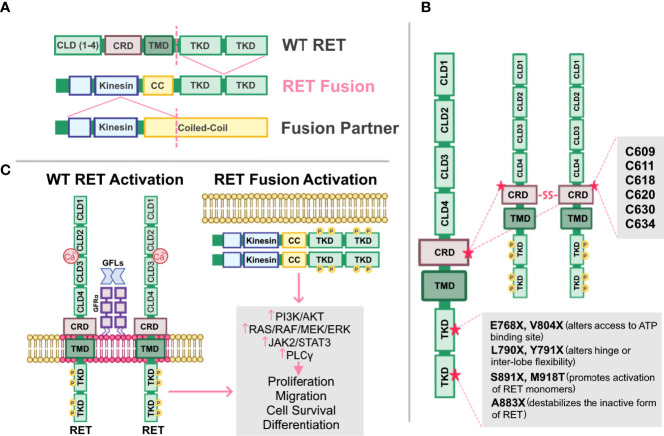
Altered RET and their mechanisms of activation. **(A)** Schematic representation of the fusion between the tyrosine kinase domain of wild-type (WT) RET receptor, and the kinesin and coiled-coil (CC) domain of a fusion partner. Dashed lines represent fusion sites. **(B)** Cysteine mutations in RET cysteine-rich domain (CRD) promote formation of intermolecular disulfide bridges, leading to constitutive dimerization and activation of RET that is GDNF-independent. Mutations in RET tyrosine kinase domains (TKD) can elicit steric conformations that regulate access to the ATP binding pocket of RET (E768X, V804X), alter hinge or inter-lobe flexibility (L790X, Y791X), promote activation of RET monomers (S891X, M918T), or destabilize the inactive form of RET (A883X), all of which confer constitutive RET activation albeit with varying activities. **(C)** Activation of either WT RET or oncogenic RET fusions can promote activation of downstream pathways, PI3K/AKT, RAS/RAF/MEK/ERK, JAK2/STAT3, and PLCγ resulting in enhanced proliferation, migration, cell survival and differentiation, ultimately promoting neoplastic growth and tumorigenesis.

RET alterations were first identified in the late 1980s with the detection of an oncogenic RET fusion in papillary thyroid carcinomas (33). Since then, additional RET rearrangements have been identified by multiple groups in a number of solid tumors (34). Oncogenic gene fusions arise from juxtaposition of two otherwise independent genes, resulting from structural rearrangements such as inversions or translocations, transcriptional reading of adjacent genes (35–37), or splicing of pre-mRNA sequences (38, 39). Most RET fusions lack the transmembrane domain, giving rise to chimeric, cytosolic proteins that exert their oncogenic influence through constitutive activation of RET kinase domain (**Figure 2A**). Since discovery of the first RET fusion in thyroid cancers, at least 13 fusion partners have been identified in papillary thyroid cancers, the most common of which are coiled-coil domain containing 6 (*CCDC6-RET*) and nuclear receptor co-activator 4 *(NCOA4*)-*RET* (40). Similarly, at least 45 RET fusion partners have been identified in lung cancers (34), with the most common being kinesin family member 5B (*KIF5B)-RET* (70-90%) and *CCDC6-RET* (10-25%). While RET fusions are detected in other cancers, such as breast cancer and salivary gland carcinomas (discussed later), RET fusions occur in these cancer types at much lower frequencies (0.16% and 3.2%, respectively) (41). Thus, RET fusions present as a clinical and therapeutic challenge in multiple tumor types (**Table 1**).

**Table 1:** Oncogenic RET alterations confer constitutive activation in multiple cancers.

Alteration type	Examples		Cancer Type	References
**RET fusions**	*CCDC6-RET (RET/PTC1)* *NCOA4-RET (RET/PTC3)*		Papillary thyroid Carcinoma	(42–48)
			Breast Cancer	(40, 49, 50)
			Colorectal Cancer	(40, 51–54)
			Salivary gland carcinoma	(55–57)
			NSCLC	(34, 40, 58–62)
	*PRKAR1A-RET (RET/PTC2)*		Papillary thyroid Carcinoma	(42, 63, 64)
	*KIF5B-RET*		NSCLC	(34, 40, 41, 51, 58, 59, 65–67)
	*MYHC13-RET*		Medullary Thyroid Carcinoma (MEN2B)	(68)
	*ETV6-RET* *TRIM27-RET* *TRIM33-RET*		Salivary Gland Carcinoma	(57, 69–74)
	*ERC1-RET*		Pancreatic carcinoma	(75)
**Germline** **mutations**	RET cysteine-rich domain	C609	Medullary Thyroid Carcinoma (FMTC, MEN2A)	(76–80)
		C611		
		C618		
		C620		
		C630		
		C634		
	RET kinase domain	E768X Y791X S891X	Medullary Thyroid Carcinoma (FMTC)	(81)
		V804X, L790X	Medullary Thyroid Carcinoma (FMTC, MEN2A)	(82, 83)
		A883X	Medullary Thyroid Carcinoma (MEN2B)	(7, 84)
		M918T	Medullary Thyroid Carcinoma (MEN2B)	(85, 86)
			Prostate Cancer	(87)

In addition to oncogenic fusions, germline mutations, such as activating point mutations, in RET are also associated with neoplasia and tumorigenesis, namely in multiple endocrine neoplasia type 2 (MEN2; described in 4.2). For example, mutations in the cysteine residues (C609, C611, C618, C620, C630, C634) within the extracellular domain of RET promotes formation of intermolecular disulfide bridges, resulting in constitutive dimerization and activation of RET that is GDNF-independent (**Figure 2B**) (76). Additionally, mutations in the RET tyrosine kinase domain elicits steric conformations that regulate access to the ATP binding pocket of RET (E768X, V804X) (81–83, 88), alters hinge or inter-lobe flexibility (L790X, Y791X), promotes activation of RET monomers (S891X, M918T) (85), or destabilizes the inactive form of RET (A883X) (7, 84, 89), all of which confer constitutive activation albeit with varying activities (90) (**Table 1**). Taken together, oncogenic RET alterations can promote constitutive activation of RET signaling, driving tumorigenesis in these affected tissues (**Figure 2C**).

### RET in papillary thyroid carcinomas

**Figure 3:**
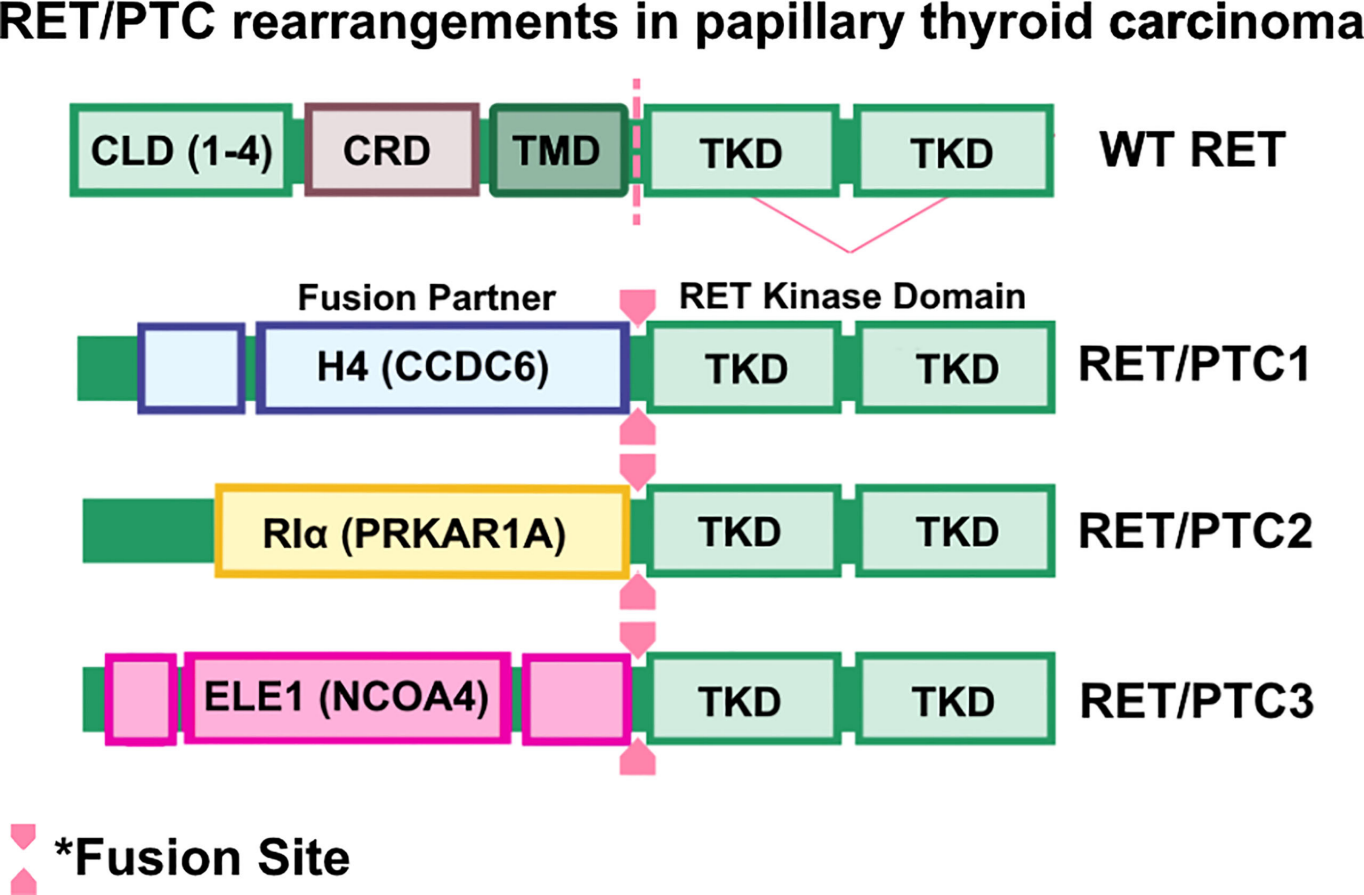
RET/PTC rearrangements in papillary thyroid carcinomas. Wild-type RET and RET rearrangements in papillary thyroid carcinoma (PTC). Coiled-Coil domain containing 6 (CCDC6)-RET, Protein Kinase CAMP-Dependent Type I Regulatory Subunit Alpha (PRKAR1A)-RET, and nuclear receptor coactivator 4 (NCOA4)-RET are frequently found in PTC cases. Oncogenic RET fusions lack the transmembrane domain (TMD) and result in chimeric, cytosolic proteins that are constitutively activated.

Papillary thyroid carcinomas (PTCs) are the most common form of differentiated thyroid cancers, the most common form of radiation exposure-induced thyroid neoplasm, and represents up to 85% of all thyroid cancer cases (91). PTC occurrence was initially associated with activating mutations of RAS and subsequent activation of the RAS/MAPK/ERK pathway (92–94). This paradigm was challenged when Fusco et al. (95) identified oncogenic RET as an unknown transforming oncogene in metastatic PTC and, while the oncogene was analyzed for homology with known kinase genes, including *ret*, the identity of this novel gene remained unclear and was therefore named ‘*PTC’.* A follow-up study by the same group determined that *PTC* is a rearranged form of RET, and rearranged RET DNA sequences were readily detectable in *PTC*-positive papillary thyroid tumors (33). These findings were corroborated by a study by Ishizaka and colleagues (96), in which they identified aberrant RET transcripts in PTC tumor samples. Further analysis by Lanzi et al. identified two additional RET rearrangements in PTC, named *RET/PTC1* and *RET/PTC2*, which were constitutively activated, tyrosine-phosphorylated, and apparently not bound to the cell membrane (42). Santoro et al. identified a third RET rearrangement, which they named *RET/PTC3* (43). In *RET/PTC1*, *RET* is fused with a then-unknown gene named *H4* (*D10S170*), which is now known as *CCDC6* (33, 97, 98), whereas *RET/PTC2* and *RET/PTC3* are products of RET kinase domain fusing to the N-terminus of *RIα* (now known as *PRKAR1A*) (63, 64) and *ELE1* (now known as *NCOA4*) (43, 99, 100) genes, respectively (**Figure 3**).

While *RET/PTC1-3* are considered unique forms of *RET/PTC* fusions, they share common features such as ubiquitous expression of the 5’ fusion partner (44, 99), altered subcellular localization of RET kinase activity (101), and constitutive activation of RET kinase arising from ligand-independent dimerization of the RET (63, 100, 101). Transgenic overexpression of *RET/PTC1* is indeed oncogenic and induces spontaneous and metastatic murine thyroid carcinomas that are histologically similar to human PTC tumors (45, 102), though whether *RET/PTC1* overexpression is an initiating factor in human thyroid carcinogenesis remained unclear. Analysis of 26 occult thyroid carcinomas, which are small papillary carcinomas with clinically apparent node metastases but are often microscopic or overlooked by ultrasonograms, determined that RET/PTC activation is found in 42% (11/26) of samples analyzed, suggesting that RET/PTC activity contributes to early papillary thyroid carcinogenesis (103). Importantly, additional studies determined that *RET* rearrangements are frequently found in individuals exposed to radiation (104), such as the children who experienced the Chernobyl nuclear plant incident (105–107), suggesting an association between radiation and emergence of *RET* rearrangements. To date, there are at least 12 oncogenic RET fusions identified in PTC (108), suggesting that RET alterations contribute significantly to initiation and progression of PTC. Further characterization of PTC tumors determined that *RET* proto-oncogene expression is primarily found in PTC subtype of thyroid cancers (109). It is important to note, however, that clonal RET/PTC rearrangements occur in 10-20% of PTC patients and may be induced by varying causative factors (110). For example, RET/PTC rearrangements are found in up to 70% of pediatric PTC cases in areas of high radioisotope contamination (46). However, Rhoden and colleagues showed that RET/PTC rearrangements are found in over 25-30% of spontaneous PTC (47, 111). Additionally, a comparative study by Zhu and colleagues determined that the method by which RET/PTC rearrangements are assessed may alter the reported frequency within the sample cohort (46). Thus, considerations must be made when assessing frequency of RET/PTC rearrangements in PTC patient samples.

### RET in medullary thyroid carcinomas

In contrast to PTC, medullary thyroid carcinomas (MTCs) are a rare type of thyroid tumor that arises in parafollicular C cells and represent 2-4% of malignant thyroid neoplasms (91, 112). While the majority of MTC tumors are spontaneous, up to 30% of MTCs are hereditary. MTCs are classified into one of the following major clinical subtypes: classical MEN2A, MEN2A with cutaneous lichen amyloidosis (CLA), MEN2A with Hirschsprung disease, FMTC, and MEN2B. Familial MTC (FMTC) subtype is characterized by occurrence of only MTC, which has relatively delayed latency and is clinically less aggressive compared to MEN2A and MEN2B subtypes (91). MEN2A was first characterized in 1961 by J.H. Sipple after identifying an association between pheochromocytoma and occurrence of a thyroid carcinoma (113). MEN2A subtype represents 70-80% of hereditary MTCs and is characterized by co-occurrence of MTC, pheochromocytoma, and/or primary hyperparathyroidism (may include Hirschsprung’s disease or CLA) (114, 115). MEN2B subtype is characterized by co-occurrence of aggressive MTC and pheochromocytoma, along with intestinal tumors, neuromas, and Marfanoid body habitus (114). The major MTC variants are summarized in **Table 2**.

**Table 2:** Major medullary thyroid carcinoma (MTC) variants.

MTC variant	Characterization	References
Classical MEN2A	Most common MTC variant MTC uniformly present PHEO or PHPT (co-occurrence is rare)	(8, 78, 114, 116, 117)
MEN2A with CLA	Co-occurrence of MTC and PHEO, and/or PHPT. Primarily harbor RET-C634 mutations	(115, 118)
MEN2A with Hirschsprung disease	Harbor RET-C609, C618, and C620 mutations HSCR associated with insufficient RET protein in gastrointestinal tract	(119–121)
FMTC	Generally less aggressive than MEN2A or MEN2B MTC only No history of PHEO or PHPT Frequently harbor RET-C634 mutations	(77, 78, 86, 122)
MEN2B	Co-occurrence of aggressive MTC and PHEO; may present with intestinal tumors, neuromas, and Marafanoid Body Habitus. Frequently harbor RET-M918T and RET-A883F mutations	(9, 18, 114)

Medullary thyroid carcinomas (MTCs) are categorized as one of five recognized variants, and MTC classification is largely dependent on co-occurrence with specific malignancies such as cutaneous lichen amyloidosis (CLA) or Hirschsprung disease (HSCR), pheochromocytoma (PHEO), or primary hyperparathyroidism (PHPT).

RET rearrangements were first associated with MTC tumors when Santoro et al. detected RET fusion products in pheochromocytomas and MTC samples (123). The following year, a study by Yamamoto and colleagues demonstrated that the mapped chromosomal location of *RET* is in close proximity with the MEN2A gene locus (116), suggesting a potential genetic correlation between *RET* expression and MEN2A occurrence. However, Donis-Keller and colleagues proposed a strict association between exon 7/8-specific RET mutations and occurrence of MEN2A and FMTC since these mutations were strictly detected in families with MEN2A and FMTC, not MEN2B (77). These findings were supported by Mulligan et. al, in which they identified germline missense mutations within the *RET* proto-oncogene in 87% (20/23 cases) of the MEN2A families but not in families without MEN2A (8). Additionally, the Mulligan et al. found that, of the 20 families with germline missense *RET* mutations, 19 of these families shared a mutation in one of the cysteine residues in the RET extracellular domain, suggesting that mutation of this cysteine residue may predispose patients to MEN2A or FMTC (8). A follow-up study analyzed 118 unrelated families with MEN2A, MEN2B, or FMTC and determined that over 95% of MEN2A families and 86% of FMTC families possessed RET-C634 mutation, which is in the immediate proximity of the transmembrane domain (78). In agreement with their earlier study (8), Mulligan and colleagues (1994) did not detect the RET-C634 mutation in the MEN2B families assessed in this study, however the frequency of RET-C634 mutation was significantly higher in MEN2A families than those with FMTC (84% vs. 50%, respectively) (78). As MEN2A patients develop hyperparathyroidism and/or pheochromocytoma that is not typically detected in FMTC, these findings suggest that the RET-C634 mutation is correlated with increased predisposition to develop MEN2A due to enhanced RET activity.

While MEN2A cases frequently acquire RET-C634 mutations, MEN2B families also share common RET mutations. RET-M918T was first detected in 1994 upon DNA sequence analysis of 9 unrelated MEN2B patients (124), which was corroborated by a separate study that detected the mutation in 34 unrelated MEN2B patients (89). RET-M918T mutation is detected in 95% of MEN2B cases (122) and elicits oncogenic signaling by stabilizing monomeric RET, enhancing binding with ATP, and is activated in absence of GDNF ligand (9, 85, 125). Additionally, RET-A883F (7, 84, 86) is a strongly activating RET mutation that is hypothesized to destabilize the inactive conformation of RET by altering the local conformation of the protein (10).

Until recently, only activating RET mutations were cited as oncogenic events in MTC. However, a 2015 case report identified an MTC patient with Myosin Heavy Chain 13 (MYHC13)-RET fusion (68), suggesting that oncogenic RET rearrangements also occur in MEN2B albeit at lower frequencies. Taken together, MTC subtypes present with unique, activating RET mutations that induce thyroid tumorigenesis.

### RET in non-small cell lung cancer

Lung cancers are the second most-commonly diagnosed cancer and the leading cause of cancer-related deaths in American men and women (126). Non-small cell lung cancer (NSCLC) constitutes up to 80-85% of all lung cancers and can further categorized into one of three major subtypes: adenocarcinoma, squamous cell carcinoma, and large cell carcinoma (127). NSCLC subtypes are frequently grouped together due to the similarities in treatment options, namely surgical resection, neoadjuvant or adjuvant chemotherapies, and immunotherapy (for a full review of NSCLC epidemiology and treatments, see Duma et. al) (128).

RET rearrangements occur in 1-2% of NSCLC cases (58, 59, 65). Since identification of the first RET fusion (*KIF5B-RET*) in lung cancer (51, 65–67), significant efforts have been made to identify additional novel RET fusions in NSCLC. While RET fusions occur in 1-2% of NSCLC cases, there are at least 48 unique RET fusion partners identified in NSCLC, which have been catalogued by Ou and Zhou (34), with the most common being *KIF5B-RET* and *CCDC6-RET*. While most NSCLC cases do not present with additional driver mutations, analysis of over 4,871 samples by Kato and colleagues identified co-occurrence of RET fusions with other genetic alterations, such as PI3K-associated genes or MAPK effector genes, in up to 82% (72/88) of NSCLC patients (41). Thus, the clinical and pathological features of RET fusion-positive NSCLC may differ from those observed in NSCLC cases driven by alternative oncogenic aberrations. Indeed, an analysis by Wang and colleagues determined that RET fusion-positive lung carcinomas had more poorly differentiated tumors compared to those with ALK or EGFR alterations, indicating that RET fusions define a unique molecular and clinicopathological subtype of NSCLC (58).

Distant metastases are of a particular concern in NSCLC, with 30-40% of cases metastasizing to the bone, lungs, brain, and adrenal glands (129). At the time of diagnosis, 10% of NSCLC patients present with brain metastases, with 30% of NSCLC patients developing brain metastases over the course of their disease (130). Baseline incidence of brain metastases in RET fusion-positive NSCLC is 27% and is independent of age, smoking status, or 5’ fusion partner status, and this proportion increases to 49% throughout the lifetime of disease (131). Thus, identifying molecular targets in brain-metastatic NSCLC and developing therapeutic compounds with blood-brain-barrier (BBB) permeability is a topic of recent investigation. Multi-kinase inhibitors (MKIs) targeting commonly altered oncogenic drivers, such as EGFR, MET, KIT, and VEGFR2, have shown moderate selectivity against altered RET in NSCLC and were thus repurposed as RET inhibitors (discussed below) (132–136), with many of these inhibitors displaying intracranial activity against brain metastases (137–139). However, a multi-institutional study by Drilon and colleagues determined that 25% of RET fusion-positive stage IV lung cancer patients presented with brain metastases at diagnosis, and less than 20% experienced intracranial response upon treatment with RET-targeting MKIs, suggesting that MKI inhibitors are inadequate for treatment of brain-metastatic lung cancers (140).

### RET alterations in other tumor types

While aberrant RET activity is primarily associated with thyroid carcinomas and NSCLC, abnormal RET signaling is also found in other tumor types, albeit at lower frequencies. This section of the review will briefly summarize the role of RET signaling in other solid tumor types and the implications of RET inhibition in these cancers.

#### Aberrant RET is actionable in breast cancers

RET is altered in ~1.2% of breast cancer cases, of which 66% are RET amplifications and 7% are activating fusions (49). As with other solid tumors types discussed here so far, the most common RET fusions detected in breast cancer are *CCDC6-RET* and *NCOA4-RET*, and their constitutive activation induces enhanced cell proliferation and survival that ultimately results in tumorigenesis (58, 141).

Early investigations of RET in breast cancer were primarily focused on estrogen receptor-alpha-positive (ER+) cases due to the prevalent co-overexpression of RET and its co-receptor, GFRα1, in a subset of ER+ breast cancers (142). Additionally, co-overexpression of RET-GFRα1 was inversely correlated with expression of basal markers and positively correlated with enhanced cell proliferation and survival of ER+ cells (143), suggesting that RET pathway activity may be a novel target in this breast cancer subtype. A study by Gattelli and colleagues determined that RET activation promotes growth, proliferation, and migration of ER+ breast cancer cells *in vitro* and *in vivo* (144), strongly suggesting that RET is actionable in ER+ breast cancers. Standard-of-care therapies for ER+ breast cancers implement ERα antagonists, such as tamoxifen and fulvestrant (145). ERα antagonists inhibit the estradiol (E2)/ERα interaction to effectively abrogate ERα signaling (146). Unfortunately, up to 50% of breast cancer patients will exhibit endocrine-resistant breast cancers or develop endocrine resistance over the course of prolonged treatment (147). Interestingly, a study from Plaza-Menacho et al. determined that RET regulates sensitivity of ER+ breast cancer cells to endocrine therapies and that activated RET promotes estrogen-independent activation of ERα (148), suggesting that RET and ERα pathway crosstalk in endocrine-resistant breast cancers.

Aromatase inhibition is another therapeutic regimen used for treatment and management of ER+ breast cancers (149). Aromatase, also known as estrogen synthase, is a critical enzyme for estrogen biosynthesis and its increased expression and/or activity can drive estrogen production to sustain growth of ER+ breast cancers (150). As observed with tamoxifen, many ER+ breast cancer cases present with aromatase-resistant breast tumors at the time of diagnosis, or develop aromatase resistance with prolonged treatment (151). In 2013, the Isacke group determined that RET signaling is hyperactivated in aromatase-resistant ER+ breast cancer and that pharmacological RET inhibition impairs GDNF-mediated aromatase inhibitor resistance (152). Additionally, RET-mediated endocrine resistance in ER+ breast cancer cells may be attributed to the finding that RET is an ER target gene and that RET and ERα stimulate unique pathways in luminal breast cancers, thus providing biological rationale for dual targeting of these two receptors to combat hormone resistance in luminal breast cancers (153–157). Thus, RET is actionable in endocrine-resistant ER+ breast cancers and these patients may benefit from combined inhibition of RET and ER pathway. However, a study done by Andreucci and colleagues determined that dual targeting of RET and aromatase yields similar preclinical benefit when compared to RET or aromatase inhibitor monotherapies (158). Moreover, while RET expression is strongly correlated with that of ER in luminal breast cancers, it lacks prognostic significance as an independent biomarker in breast cancer patient tissue (142), suggesting that additional studies are required to determine if targeting RET will yield clinical benefit in luminal breast cancer patients.

Of all breast cancer subtypes, HER2-enriched and triple-negative breast cancers (TNBCs) are the most aggressive subtypes with the highest propensity for metastases (159). RET protein is expressed in HER2-enriched breast cancers and triple-negative breast cancers (153). While trastuzumab is one of the frontline therapies administered to HER2-enriched breast cancers, TNBC patients receive neoadjuvant or adjuvant chemotherapy and surgical resection (160). Despite initial response observed in HER2-positive breast cancer patients receiving trastuzumab, they eventually present with acquired trastuzumab resistance that is difficult to treat (161, 162). A recent report associates trastuzumab resistance with activation of a RET-HER2 signaling axis, whereas trastuzumab-naïve cells rely on a RET-SRC-HER2 signaling (161), suggesting that trastuzumab resistance may be a consequence of Src-independent signaling in HER2-enriched breast cancers that have concurrent RET activation. Similarly, TNBCs lack effective treatments that yield long-term clinical benefit. While RET activity has not been directly identified as a pro-tumorigenic kinase in TNBC, multiple studies demonstrate that MKIs can target RET to inhibit TNBC growth and proliferation *in vitro* and *in vivo.* For example, vandetanib can target RET, as well as, vascular endothelial growth factor receptor 2 (VEGFR-2) and epidermal growth factor receptor (EGFR), but exhibits potent anti-tumor effects against TNBC patient-derived xenografts with RET hyperactivity (163), suggesting RET inhibition as a novel treatment modality in TNBC patients. Moreover, recent reports suggest that RET may regulate breast cancer metastases. A recent study identified increased expression of RET in breast cancer brain metastases (BCBM) relative to matched primary breast tumors, and that treatment of these BCBM tissues with MKI cabozantinib reduces RET activation, inhibits BCBM growth, and concurrently induces apoptosis (164). In summary, these findings suggest that RET is actionable in breast cancers, especially those of HER2-enriched and triple-negative subtype.

In addition to oncogenic RET alterations, co-overexpression of wild-type RET and its co-receptor, GFRα1, may also contribute to breast tumorigenesis. RET and co-receptor GFRα1 are overexpressed in 25-75% of breast cancer cases (50). Esseghir and colleagues (2007) demonstrated that co-overexpression of RET and GFRα1 promoted cell proliferation and survival of luminal MCF7 cells in response to stimulation with GDNF, and that GDNF is overexpressed by stromal cells in response to the pro-inflammatory signals within the tumor microenvironment (143). A later study by Boulay et al. (2008) determined that RET and ERα pathway share a functional crosstalk in luminal breast cancer cells and RET expression can be enhanced upon stimulation of the ERα pathway (153), suggesting that RET-positive luminal breast cancers may benefit from RET inhibition. Therefore, in addition to the ~1.2% of breast cancer cases harboring RET alterations (49), breast cancer patients overexpressing RET-GFRα1 may also benefit from RET-targeted therapies.

#### RET in salivary gland carcinomas

The World Health Organization (WHO) classifies salivary gland tumors into more than 30 malignant and benign subtypes based on cytology rather than the site of the originating tumor (69, 165, 166). Genomic profiling salivary duct carcinomas (SDCs) reveals that RET is frequently altered in this tumor type, with three clinical cases presenting with RET fusion products, *CCDC6-RET* or *NCOA4-RET* (55). While this was a retrospective study, the authors also described that patients with *NCOA4-RET* fusion-positive SDC tumors responded well to cabozantinib, and these patients experienced reduction in chest wall lesions, and improved palpable neck relapse. Intraductal salivary gland carcinomas (ISGCs) are controversially linked to SDCs, with some arguing that these malignancies should be classified as two separate subtypes. ISGCs were first characterized in 1983 as a benign mass that was comprised entirely of intraductal components (167), which was a stark contrast to the infiltrating salivary duct carcinomas first described in the late 1960s (168–170). ISGCs are histologically unique from low grade-intraductal carcinomas (LG-ICs), which were first described by Delgado and colleagues in 1996 as a benign mass that resembled mammary ductal hyperplasia (171). Considered as a rare salivary gland tumor type, LG-ICs typically originate in the parotid gland, are more commonly found in the female population, and are generally asymptomatic (172). Next-generation RNA sequencing of 23 LG-IC samples identified one case with *NCOA4-RET* fusion, with a total of 47% of ICs presenting with some form of RET rearrangement (56), suggesting that RET is an early driver of salivary gland carcinogenesis. A similar study reported that 8/17 (47%) cases of salivary gland ICs had RET rearrangements (70). Furthermore, several studies have identified oncogenic RET fusions in varying subtypes of salivary gland carcinomas, namely *ETV6-RET* in mammary analogue secretory carcinomas (71–73, 173), *NCOA4-RET* in intercalated duct-line SDCs (57, 70, 173), *TRIM27-RET* fusions in mixed intercalated duct-like and apocrine types of SDC (57, 70), and *TRIM33-RET* fusions were recently detected in oncocytic intraductal carcinoma (74). Taken together, these findings support RET as a potential therapeutic target in multiple subtypes of salivary gland carcinoma.

#### RET in prostate cancer tumorigenesis

Prostate cancer is the most commonly diagnosed cancer and second-leading cause of cancer-related deaths in American men (126). While early stage prostate cancers do not usually present with clear symptoms, those that arise are similar to those found in benign prostatic hyperplasias and can include frequent urination, hematuria (blood in urine), dysuria (pain during urination), fatigue, and bone pain (174). Early stage prostate cancers are classified as prostatic intraepithelial neoplasia (PIN) and show enhanced epithelial cell proliferation within benign prostatic acini (175). As PIN satisfies many of the classification requirements of premalignant disease, high-grade PIN is considered the precursor to prostate cancer (176, 177). Malignant prostate cancers progress from PIN to prostatic adenocarcinoma, and eventually to metastatic prostate cancer that may or may not be castration-resistant (178). Prostate cancer screenings include prostate exams as well as blood tests for prostate-specific antigen (PSA) (175), though whether PSA testing improves disease outcome remains to be confirmed.

Overexpression of wild-type RET in prostate cancer was first demonstrated by Robinson and colleagues (179) using a prostate cancer xenograft model. A follow-up study showed that RET protein is overexpressed in high-grade PIN and prostate cancers relative to benign prostatic tissue, and that RET protein expression was positively correlated to the tumor Gleason score (180), suggesting that RET overexpression may contribute to malignant progression of prostate cancers. Concurrent studies by Ban et al. revealed that nearly 19% (61/325) of prostate cancer tissues examined exhibit tumor tissue-specific RET overexpression (181) and that RET expression is critical for prostate cancer growth *in vivo* (181). However, the increase in RET activity within prostate cancers may also be attributed to increased secretion of GDNF by prostatic stromal cells (182) or GFRα1 by peripheral nerves within the prostate (181, 183, 184), ultimately promoting perineural invasion of prostate cancers.

As discussed in sections 4.2 and 4.3, aberrant RET activation is frequently found in neuroendocrine tumors such as papillary and medullary thyroid cancers. A 2018 case report described a 20-year-old MTC patient that presented with an activating point mutation in RET (RET-M918) four years after total thyroidectomy and presented with prostatic adenocarcinomas twenty years later (87). As the prostate contains epithelial and neuroendocrine cells that express RET protein, this case report suggests an association between RET hyperactivity in neuroendocrine cells and initiation of prostatic neoplasia. In 2020, VanDeusen and colleagues reported that aggressive, androgen-independent prostate cancers can transdifferentiate into neuroendocrine prostate cancers (NEPC), which exhibit tumorigenic RET activity, though this can be suppressed using selective RET inhibitors (185). It is important to note, however, that while RET overexpression is frequently found in prostate cancers (180, 181), RET alterations are found in 1-2% of prostate cancer cases (186). Taken together, these findings suggest that aberrant RET activity may be actionable in prostate cancers, including those associated with neuroendocrine malignancies.

#### Aberrant RET in colorectal cancers

Colorectal cancer (CRC), also known as colon, bowel, or rectal cancer, represents 7-10% of all cancer cases and is one of the leading causes of cancer-related deaths in American men and women (126, 187). CRC originates from epithelial cells lining the colon and gastrointestinal tract and begin as benign polyps that grow into large polyps, which develop into adenoma, followed by carcinoma (188). Metastatic CRCs frequently metastasize to the liver, lungs, bones, brain, liver, and peritoneum (189).

Ishizaka and colleagues identified a second form of activated RET, *ret-*II, in human sigmoid colon cancer, which lacked the coding region for the transmembrane domain found in wild-type RET (190). A follow-up study determined that *ret-*II is likely a gene fusion as the sequences preceding the kinase domain of *ret-*II were of unknown origin (191). Large-scale genomics analyses demonstrate that *RET* fusions, including *CCDC6*-*RET* and *NCOA4*-*RET*, are found in 0.2% (6/3117) of CRC cases (52). Analyses of metastatic CRC (mCRC) cases reveal that RET-rearranged mCRCs have poorer overall survival and may benefit from pharmacological RET inhibition (53). Indeed, patient-derived CRC tumor cells harboring *NCOA4-RET* fusion were sensitive to the treatment with vandetanib, a multi-kinase inhibitor with non-selective RET activity (54).

Despite the evidence supporting an oncogenic role for RET alterations in CRC, the role of RET in this cancer type remains controversial. A 2013 study by Luo et al. reported enhanced methylation, and therefore downregulation, of RET in CRC samples, suggesting a tumor suppressive function for RET in the CRC samples analyzed (192). The potential tumor suppressive function of RET is further supported by a 2021 study in which immunohistochemical analyses demonstrate a reduction in RET protein expression in CRC tissue when compared to adjacent normal tissue (193), suggesting an inverse correlation between RET expression and CRC development. While the opposing paradigms of RET function may present challenges in the treatment of RET-altered CRC tumors, further analyses are required to determine whether tumor-suppressive or oncogenic RET occurs in specific subpopulations of CRC tumors.

#### RET in pancreatic and ovarian cancers

RET is altered and potentially actionable in other solid tumor types, albeit at low frequencies. RET alterations occur in ~1.9% of pancreatic cancers (41) whereas wild-type RET, co-receptor GFRα1, and GDNF are overexpressed in 50-70% of pancreatic cancer cases (194, 195). Additionally, Ceyhan and colleagues demonstrated that RET activation by another GFL, Artemin, can promote invasion and migration of pancreatic cancer cells (196). This finding was supported by a later study in which enhanced RET overexpression promotes pancreas tumorigenesis and perineural invasion using a transgenic mouse model of pancreatic cancer (197), suggesting that enhanced activity of wild-type RET is adequate to promote metastatic pancreatic cancer progression. Moreover, enhanced secretion of GDNF by cells within the tumor microenvironment stimulates RET activity in pancreatic tumor cells, thus exerting a paracrine effect on pancreatic cancer cells to drive perineural invasion (197, 198). While RET alterations are rare in pancreatic cancers, exemplified by report of the first RET fusion-positive pancreatic cancer patient in 2021 (75), patients with aberrant RET pathway activity may benefit from RET-targeted therapies. In addition to pancreatic cancers, RET is altered in ~1.2% of ovarian cancers (41). Oncogenic RET variants have been recently identified in epithelial ovarian carcinomas, which were sensitive to treatment with vandetanib, an MKI with non-selective RET activity (199). However, additional studies are required to determine the therapeutic utility of RET inhibition in ovarian cancers.

## Multi-kinase inhibitors with non-selective RET activity

Due to the numerous studies implicating aberrant RET kinase activation in varying solid tumors, pharmacological inhibition of RET has become a promising new treatment modality in patients with enhanced RET activity. Targeting RET activity began with repurposing of MKIs that were initially designed to target other kinases, but showed moderate selectivity to RET. This portion of the review will summarize the MKIs with anti-RET activity.

### Cabozantinib

Cabozantinib (XL184, sold as Cometriq ^®^ and Cabometyx ^®^) is an orally available ATP-competitive MKI that can inhibit c-MET, VEGFR2, and a number of other kinases including RET, FLT3, TRK, and KIT (200). Cabozantinib effectively inhibits cell proliferation, growth, migration, and invasion *in vitro*, and promotes tumor cell and endothelial cell apoptosis *in vivo* in a number of cancer cell lines (200). Cabozantinib was well-tolerated in a number of clinical trials including MTC (201) and renal cell carcinoma (202). Cabozantinib received FDA approval in 2012 for treatment of MTC, as second line of treatment in kidney cancer in 2016, treatment of renal cell carcinoma in 2017, and again in 2019 for treatment of hepatocellular carcinoma. A phase II clinical trial (NCT01639508) reported that RET-fusion positive lung cancer patients receiving cabozantinib (60mg, p.o. daily) demonstrated partial response (28%; 7/25 patients) and fortifies the notion that RET is actionable in lung cancers (132, 203). Similarly, daily oral administration of cabozantinib (140mg) enhanced overall survival (26.6 vs. 21.1 months in treatment vs. placebo group, respectively) and progression-free survival in phase III clinical trial (NCT00704730) for treatment of metastatic MTC cases harboring RET-M918T with an acceptable safety profile (204, 205). The overall ORR for patients who received cabozantinib was 28%, but increased to 34% when assessing only patients with RET-M918T mutations. While data is limited for use of cabozantinib in treatment of central nervous system (CNS) tumors, such as BCBM, cabozantinib penetrates the BBB and may prove to be effective in targeting brain malignancies arising from RET-altered cancers (137, 206). While cabozantinib received FDA approval for treatment of MTC (207), ongoing clinical trials aim to assess cabozantinib safety and efficacy in RET fusion-positive NSCLC (NCT01639508, NCT04131543).

### Lenvatinib

Lenvatinib (E7080; Lenvima ^®^) is an oral ATP-competitive MKI with activity against FLT-1, FLT-4, KIT, FGFR1, PDGFR, VEGFR1-3, and can inhibit angiogenesis and growth of SCLC and breast cancer cells *in vitro* and *in vivo* (208–211). When evaluated in RET fusion-positive preclinical models, lenvatinib effectively inhibits RET fusion kinases and RET pathway activity *in vitro*, and inhibits growth of RET fusion-positive tumor cells *in vivo* (212, 213). While a phase II clinical trial (NCT01877083) determined that once-daily oral administration of lenvatinib (24mg) induces relatively low response in RET fusion-positive NSCLC patients (ORR 16%, 4/25 patients), this effect was adequate to improve the median progression-free survival (214). Lenvatinib received FDA approval in 2015 for treatment of differentiated thyroid cancer (215) and 2018 for treatment of unresectable hepatocellular carcinoma (216). Lenvatinib has also received FDA approval as part of combination therapies in renal cell carcinoma (with mTOR inhibitor everolimus) (217) and endometrial carcinoma (with anti-PD-1 antibody pembrolizumab) (218).

### Vandetanib

Vandetanib is an orally available inhibitor of KDR/VEGFR-2 tyrosine kinase with moderate activity against FLT-1/VEGFR-1, EGFR, and PDGFRβ (219, 220). Vandetanib potently inhibits VEGF signaling and angiogenesis, resulting in reduced growth of varying tumor cell xenografts, namely human lung, prostate, breast, ovarian, and colon cancer cells, with little to no acute toxicity (221). Within the same year, Carlomagno and colleagues showed that vandetanib potently inhibited kinase activity of RET/PTC, RET/MEN2A, and RET/EGFR in transformed NIH3T3 cells, reverted the morphological phenotype of transformed cells to that of parental cells, and inhibited anchorage-independent growth (222). Vandetanib selectivity for RET was further validated in a *D. melanogaster* model of MEN2 syndromes and papillary thyroid carcinomas, suggesting that vandetanib can inhibit multiple RET isoforms to treat RET-dependent carcinomas (223). However, a follow-up study by Carlomagno et al. identified RET-V804L or RET-V804M gatekeeper mutations which confer resistance to vandetanib (83), suggesting that vandetanib may not yield clinical efficacy in cases with RET gatekeeper mutations. Clinical trials determined that vandetanib is well-tolerated when administered once daily (300mg, p.o.), elicits anti-tumor response in patients with advanced or metastatic hereditary MTC (224, 225), demonstrated improved therapeutic efficacy, and was predicted to enhance progression-free survival (PFS) in advanced MTC (19.3 months vs. 30.5 months in placebo vs. treatment, respectively) in phase III clinical trial (NCT00410761) (226). As with other MKIs, vandetanib can inhibit many kinases (220–223), thus the anti-tumor effects observed in MTC may not strictly be due to inhibition of RET kinase activity. Nevertheless, vandetanib received FDA approval for treatment of non-resectable advanced or metastatic MTC in 2011 (227).

### Sorafenib

Sorafenib (Nexavar ^®^) is an orally available ATP-competitive inhibitor of Raf kinase (228) with activity against other kinases, such as BRAF, VEGFR-2 and -3, and PDGFRβ, to impede tumor growth and angiogenesis *in vivo* (229). Subsequent studies illustrated that sorafenib inhibits activity of wild-type RET and oncogenic altered RET with some degree of selectivity to abrogate PTC tumor cell proliferation and growth *in vitro* and *in vivo* (230–232). Moreover, RET inhibition by sorafenib is not affected by the RET-V804M gatekeeper mutation, suggesting that sorafenib may yield clinical benefit in MTC patients bearing RET-V804 mutation (231). In addition to being an ATP-competitive inhibitor, Plaza-Menacho and colleagues demonstrated that sorafenib treatment promotes degradation of RET protein independently of proteosomal targeting (231), which suggests a secondary mechanism by which RET activity is depleted upon treatment with this inhibitor. Interestingly, a 2012 study by Mao et al. corroborated the finding that sorafenib affects RET expression and concurrently alters VEGFR2 expression, although these findings are cell-line dependent and require additional mechanistic studies (233). Sorafenib received FDA approval in 2005 for treatment of advanced renal cell carcinoma (234), and was subsequently approved for treatment of unresectable hepatocellular carcinomas (235) and iodine-refractory, metastatic differentiated thyroid cancers (236). Interestingly, an exploratory study by Horiike and colleagues determined that NSCLC patients do not exhibit response to sorafenib (136), suggesting that RET fusion-positive NSCLC patients may not gain clinical benefit from this compound, though this requires additional testing in RET-altered NSCLC patients and other RET-altered cancers.

### Regorafenib

Regorafenib (BAY 73-056; Stivarga ^®^) is an orally bioavailable ATP-competitive inhibitor that targets VEGFR1-3, TIE2, PDGFRβ, KIT, and RET *in vitro* and *in vivo*, effectively functioning as an anti-angiogenic, anti-tumorigenic, and pro-apoptotic compound (237). Regorafenib is well-tolerated when administered as a single agent and elicited a partial response or stable disease in patients with advanced or non-resectable solid tumors (238–240), though renal cell carcinoma patients experienced adverse events with prolonged treatment (241). Chen and colleagues illustrated that regorafenib potently inhibits RET activity and RET-mediated PI3K/AKT signaling in neuroblastoma cells to inhibit their growth *in vitro* and *in vivo* (242). Additionally, regorafenib targets the RET-Src signaling axis to inhibit JAK1/2-STAT1 and MAPK signaling and concurrently inhibits PD-L1 expression, suggesting inhibition of RET-Src axis may impact immune response to tumor cells *in vivo* (243). While regorafenib is currently in numerous clinical trials for use in various cancer types (ClinicalTrials.gov), it has not entered trials for use in RET-altered cancers.

### Sunitinib

Sunitinib (SU11248; Sutent ^®^) was developed in 2003 as an orally bioavailable ATP-competitive inhibitor of VEGFR, PDGFR, KIT, and FLT3 receptor tyrosine kinases, and effectively inhibits tumor growth of multiple cancer cell types, namely breast, NSCLC, colon cancer, glioma, and melanoma when administered daily as a single agent (244–247). As a VEGF inhibitor, sunitinib effectively inhibits angiogenesis of tumor cells, reduces endothelial cell migration, and inhibits tubule formation *in vivo* (248). Sunitinib is potent against PTC tumors harboring *RET/PTC3* fusions and effectively inhibits downstream MAPK signaling (249). While sunitinib received FDA approval in 2006 for treatment of GI stromal tumors and advanced renal cell carcinomas, a pilot clinical trial to test safety and efficacy of sunitinib (50mg, p.o. daily) in RET fusion-positive solid tumors (NCT02450123) was placed on hold in 2020 and is of unknown status at the time of this publication.

### Alectinib

Alectinib (CH5424802; Alecensa ^®^) is an orally bioavailable, small molecule competitive ATP inhibitor developed to inhibit ALK alterations and gatekeeper mutations while sparing MET activity (250). Alectinib effectively reduces ALK activation *in vitro* and *in vivo*, inhibits activation of STAT3, PI3K/AKT, and MAPK pathways, inhibits growth of tumors harboring ALK alterations (250), and even inhibited ALK-L1196M, a gatekeeper mutation that conferred resistance to earlier generations of ALK inhibitors (251). Alectinib was well-tolerated and highly active in patients with NSCLC patients with ALK alterations (252). Kodama et al. showed that alectinib inhibits wild-type RET and the constitutively active RET-M918T NSCLC cells *in vitro* and *in vivo*, and exhibits selectivity against RET with gatekeeper mutations (V804L and V804M) (134). However, while clinical assessment determined that alectinib was well-tolerated in treatment-naïve patients, twice-daily oral administration of alectinib (600mg) exerts minimal activity in RET fusion-positive NSCLC patients (133) and yielded an overall response rate (ORR) of 4% (1/25 patients) and PFS of 3.4 months (135). These findings were corroborated by a recent clinical trial (NCT03131206) in which RET fusion-positive thyroid cancer and NSCLC patients showed minimal to no response to alectinib. Alectinib received accelerated FDA approval in 2015 for treatment of patients with ALK fusion-positive metastatic NSCLC that were unresponsive to crizotinib, an ALK and ROS1 inhibitor (253), and is currently in clinical trials to test efficacy against other solid tumors alone (NCT04116541, NCT02314481, NCT03194893) or in combination with other compounds (NCT03944772).

## Selective RET inhibitors

While MKIs show some selectivity in targeting RET in preclinical models, many of these inhibitors have suboptimal responses upon clinical administration to RET fusion-positive patients. A retrospective multi-center analysis by Gautschi et al. found that, of the 165 NSCLC patients with RET alterations who received MKIs, only partial responses were observed in patients who received cabozantinib (37%), sunitinib (22%), or vandetanib (18%) (254). This finding suggests that, despite their activity against RET, MKIs yield suboptimal clinical benefit with prolonged administration to patients. Earlier MKIs that inhibit RET are only moderately potent against RET, and cause toxicity due to stronger selectivity against other kinases, such KDR/VEGFR2, due to structural similarities with RET. Furthermore, MKIs are not selective against gatekeeper mutations that may elicit secondary resistance. Together, these findings have warranted development of selective RET inhibitors with the aim of explicitly inhibiting aberrant RET activity while sparing other kinases. This section summarizes RET-selective inhibitors. (Ongoing clinical trials against RET-altered cancers are summarized in **Table 3**).

**Table 3:** Ongoing clinical trials of RET-targeting inhibitors.

Drug class	Drug Name	Clinical Trial Identifier	Patient Cohort	Pts	Regimen	Primary Endpoint(s)	Phase
Multi-kinase inhibitor	Cabozantinib	NCT01639508	RET-altered NSCLC	86	60mg daily (28-day cycle)	ORR (203)	II
		NCT04131543	RET-altered NSCLC	25	20, 40, or 60mg daily (28-day cycle)	ORR	II
	Alectinib	NCT03194893	Continued access to alectinib	200	600mg twice daily	Safety/efficacy endpoints; DFS	III
First-generation RET inhibitor	Pralsetinib	NCT03037385	Solid tumors with or without RET alterations	589	Phase 1: dose escalation; Phase 2: 400mg once daily	Phase I: MTD, AE; Phase II: ORR, AE (255, 256)	I/II
		NCT04760288	Safety and efficacy compared to standard-of-care therapy in RET-altered MTC	198	400mg once daily	PFS	III
		NCT04222972	Safety and efficacy compared to standard-of-care therapy in RET-altered NSCLC	226	400mg once daily (257)	PFS	III
	Selpercatinib	NCT03157128	Solid tumors with or without RET alterations	989	Phase I: dose escalation; Phase II: 160mg (RP2D) twice daily	Phase I: MTD, RP2D (258) Phase II: ORR	I/II
		NCT04280081	RET-altered solid tumors in Chinese patients	77	160mg twice daily (28-day cycle)	ORR (259)	II
		NCT04268550	RET fusion-positive NSCLC (late-stage or recurrent)	124	Twice daily oral administration (28-day cycle)	ORR	II
		NCT04320888	Pediatric patients with RET-altered solid tumors, lymphomas, or histiocytic disorders	49	Twice daily (28-day cycle until disease progression or unacceptable toxicity)	ORR	II
		NCT04194944	Selpercatinib compared to chemotherapy± pembrolizumab in advanced or metastatic RET fusion-positive NSCLC	250	160mg, twice daily (21-day cycle) (260)	PFS	III
		NCT04759911	Neoadjuvant administration prior to resection of RET-altered thyroid cancers	30	(28-day cycle; up to 7 cycles)	ORR	II
		NCT04211337	Safety and efficacy compared to standard treatment of non-resectable RET-altered MTC	400	Oral administration	PFS	III
		NCT03899792	Pediatric patients with RET-altered solid tumors or primary CNS tumors	100	Phase I: dose escalation; Phase II: dose expansion to determine RP2D equivalent to adult recommended phase 2 dose	Safety; DLT, ORR (261)	I/II
		NCT04819100	Adjuvant administration following surgery or radiation in NSCLC	170	Oral administration	EFS	III
Next-generation RET inhibitor	HM06	NCT04683250	Adults with RET-altered solid tumors	202	Phase I: dose escalation starting at 20mg twice daily (21-day cycle); Phase 2: dose expansion (RP2D; 21-day cycle)	Phase I: MTD Phase II: ORR	I/II
	TPX-0046	NCT04161391	Adults with RET-altered advanced solid tumors	462	Phase I: dose escalation; Phase II: dose expansion to determine RP2D	DLT, RP2D, ORR	I/II

A summary of ongoing clinical trials of MKIs with non-selective RET activity, and first- and next-generation selective RET inhibitors. Published information regarding patient cohort, number of patients (Pts), treatment regimen, and primary endpoint are included. ORR: overall response rate; DFS: disease-free survival; EFS: event-free survival; MTD: maximum tolerated dose; AE: adverse event; RP2D: recommended phase II dose; DLT: dose-limiting toxicity.

### Agerafenib

In an effort to overcome the suboptimal clinical benefit of MKIs in RET-altered cancers, agerafenib (CEP-32496; RXDX-105) was developed as an orally available, ATP-competitive, selective inhibitor of RET and BRAF (262). Preclinical assessment in multiple RET-altered cancer types showed that RXDX-105 potently inhibits wild-type and altered RET more potently and selectively than MKIs with non-selective RET activity, and elicited rapid response in the primary tumor and brain metastases of a RET fusion-positive lung cancer patient (262). These findings strongly suggested RXDX-105 as a promising new anti-cancer compound with BBB penetrance. However, a phase I/Ib trial (NCT01877811) revealed that daily oral administration of agerafenib (275mg) does not elicit response in RET fusion-positive NSCLC patients (0/20) and conferred multiple treatment-related adverse events such as diarrhea, fatigue, and hypophosphatemia (263). Ultimately, this clinical trial was discontinued due to lack of clinical response.

### Selpercatinib

Selpercatinib (LOXO-292, LY3527723; Retevmo ^®^) is an orally available RET inhibitor that competitively binds to its ATP binding pocket (264). *In vitro* studies reveal that selpercatinib selectively inhibits both wild-type and altered RET, spares KDR/VEGFR2 activity, and selectively targets RET-altered cancer cells (264). Selpercatinib also abrogates tumor growth of lung cancer and MTC xenografts in a dose-dependent manner *in vivo*, highlighting its therapeutic utility in RET-dependent cancers. Further *in vitro* and *in vivo* characterization confirms that selpercatinib is selective against RET alterations, including fusions and activating mutations, is well-tolerated upon daily oral administration, and elicited rapid clinical response in two patient cases (MTC and NSCLC) (265). Selpercatinib entered a phase I/II clinical trial (NCT03157128) in patients bearing RET alterations (fusions, mutations, and other alterations). This clinical trial, LIBRETTO-001, remains ongoing but recently reported an objective response of 64% (67/105 patients), with treatment-naïve patients reporting 85% objective response (33/39 patients) with daily oral administration of 20-240mg selpercatinib (266). Since lung cancers frequently metastasize to the brain, effective therapeutic agents should also display BBB penetrance. In the LIBRETTO-001 trial, the authors reported that NSCLC patients with CNS metastases had over 82% objective intracranial response rate (18/22 patients), with two patients undergoing complete response, indicating that selpercatinib can penetrate the BBB to act upon brain metastases of RET-altered lung cancers (265, 267). The same clinical trial also determined an objective response of 69-79% in MTC patients bearing RET alterations (268). Many of these patients (NSCLC or MTC) reported improved or maintained quality of life during treatment with selpercatinib (269, 270). Taken together, these results indicate that selpercatinib elicits durable efficacy, including intracranial activity, with primarily low-grade toxicity effects in patients with RET-altered cancers. Selpercatinib received accelerated approval from the FDA in 2020 for patients with metastatic RET fusion-positive NSCLC, and patients with RET-mutant aggressive or metastatic MTC (271, 272), making it the first FDA approval for RET fusion-positive NSCLC and MTC. In addition to the LIBRETTO-001 trial, ongoing clinical trials are underway to test efficacy of selpercatinib in multiple tumor types, such as pediatric solid tumors (NCT03899792, NCT04320888) and advanced NSCLC (NCT04268550).

### Pralsetinib

As with selpercatinib, pralsetinib (BLU-667; Gavreto ^®^) is an orally available, ATP-competitive inhibitor that selectively inhibits wild-type and altered RET while sparing KDR/VEGFR2 activity (273). Pralsetinib entered Phase I/II clinical trial in 2017 (NCT03037385) to assess safety and efficacy in patients with RET fusion-positive thyroid and lung cancers (274). This clinical trial, entitled ARROW, is a multi-cohort, open-label, first-in-human study that involved 75 sites in 11 countries. An ORR of 61% was observed in NSCLC patients who previously received platinum chemotherapy (53/87 patients), 70% (19/27) in treatment-naïve patients, with a small percentage undergoing complete response (255). Additionally, pralsetinib showed robust intracranial activity in 78% (7/9) of NSCLC patients with CNS metastases and mitigated progression of new CNS lesions, suggesting that pralsetinib crosses the BBB to act on brain metastases (274). Pralsetinib was granted accelerated approval by the FDA in 2020 for adults with metastatic RET fusion-positive NSCLC, and patients with metastatic RET-mutant MTC that may or may not be radioactive iodine-refractory (275). While pralsetinib is well-tolerated in patients included within the ARROW clinical trial, a recent report characterized leptomeningeal progression in a NSCLC patient despite durable extracranial response to pralsetinib, though this patient exhibited clinical CNS response following selpercatinib treatment (276), suggesting that selpercatinib may improve on the intracranial therapeutic efficacy of pralsetinib. In addition to ARROW, another ongoing clinical trial aims to assess efficacy of pralsetinib compared to first-line treatments of advanced NSCLC (NCT04222972), as well as test efficacy compared to standard-of-care therapies in NSCLC (NCT04222972) and MTC (NCT04760288).

## Resistance to first-generation RET inhibitors and development of next-generation compounds

While selpercatinib and pralsetinib effectively and selectively inhibit RET in preclinical and clinical settings, recent publications report novel acquired resistance to selpercatinib or pralsetinib with prolonged treatment as well as novel genetic mutations. Analysis of circulating tumor cell DNA, isolated from RET fusion-positive NSCLC or MTC patients who progressed after initial response to selpercatinib, revealed the emergence of three mutations: G810R, G810S, and G810C within the RET solvent front, which are predicted to sterically hinder binding of selpercatinib to RET fusions (277). These findings were corroborated by other studies where, in addition to detection of G810 solvent front mutations, off-target MET (278) or KRAS amplifications were detected in RET fusion-positive NSCLC patients who received either selpercatinib or pralsetinib, albeit at low frequency (60). Additional mutations in the hinge region of RET (Y806) are strongly associated with resistance to selpercatinib or pralsetinib (279). Moreover, Subbiah et al. identified novel *KHDRBS1-NTRK3* and *KIF5B-RET* rearrangements in selpercatinib-resistant tumor samples not previously detected in primary tumor biopsy (280). It is crucial to note that mutation-driven resistance to RET inhibition may vary between inhibitors. For example, Shen and colleagues illustrate that the RET-L730V/I roof mutation confers resistance to pralsetinib, but not selpercatinib (281), which suggests unique mechanisms of binding and inhibition between these two small molecule RET inhibitors. Taken together, while the current generation of selective RET inhibitors elicits potent response in NSCLC and MTC patients, acquired resistance may arise in patients with prolonged administration.

To address the observed acquired resistance to first-generation RET inhibitors, next-generation RET inhibitors are currently being tested in Phase I/II clinical trials. BOS172738 is an investigational compound with potent selectivity against RET, spares VEGFR2 activity, overcomes the RET-G810 resistance mutation, and displays a favorable safety profile when administered as a single-agent to patients with RET fusion-positive NSCLC or MTC tumors (282). Another investigational compound, TPX-0046, showed potent activity and selectivity in RET fusion-positive cancer cells *in vitro* and *in vivo*, and can overcome RET-G810 resistance mutations (283). While TPX-0046 cannot circumvent the V804 gatekeeper mutation, a Phase I/II clinical trial (NCT04161391) is ongoing in patients with advanced solid tumors harboring RET alterations. HM06, another investigational compound, selectively inhibits RET across multiple tumor types *in vitro* and *in vivo*, circumvents RET-V804X gatekeeper mutation and RET-G810X resistance mutations (284, 285), and is currently in Phase I/II clinical trials in patients with RET-altered tumors (NCT04683250). Lastly, LOX-18228 is a novel compound that potently inhibits RET, RET fusions, and can even inhibit RET-V804X and RET-G810X mutations singly or in tandem (286), suggesting that LOX-18228 may be effective in targeting tumors with resistance to first-generation RET inhibitors. LOX-18228 is expected to enter Phase I clinical trials in 2022 (286).

## Concluding remarks

Over the last three decades, cumulative studies have established an important role that RET plays in multiple cancer types. Identification of activating RET alterations and of oncogenic RET fusions in solid tumors has led to the repurposing of MKIs in an effort to inhibit aberrant RET activity (133–136, 139, 203, 212, 230–232, 242, 249). However, these MKIs show suboptimal clinical benefit in patients and warranted the development of selective RET inhibitors that not only inhibit RET activity, but also serve to overcome RET alterations that confer resistance to RET-targeting MKIs. Selpercatinib and pralsetinib are the only selective RET inhibitors FDA-approved for treatment of RET fusion-positive lung and thyroid cancers (271, 275) and show potent intracranial activity (267, 274). While these inhibitors show significantly improved clinical benefit, recent reports describe disease progression in some patients after prolonged treatment with selpercatinib or pralsetinib, some of which are attributed to novel RET mutations that confer this acquired resistance (60, 277–280). These findings strongly support development of second generation selective RET inhibitors that target novel RET mutations as well as the studied oncogenic RET alterations described herein. Moreover, the role of RET in distant metastases remains to be fully examined. While selective RET inhibitors show potent activity in brain metastases of lung and thyroid cancer patients (267, 274), the emergence of therapeutic resistance to RET inhibitors suggests activation of additional or alternative pathways that circumvent pharmacological inhibition of RET. In summary, pharmacological inhibition of RET proto-oncogene is a promising new treatment modality in solid tumors, though additional efforts should be placed in understanding acquired therapeutic resistance to RET inhibition.

## Author contributions

Designed the manuscript, AR. Selected the reviewed literature, AR. Compiled the review tables and figures, AR and MN. Wrote the manuscript, AR. Contributed literature search, HWL. Table preparation, AR, MN. Manuscript writing and editing, AR, MN, and H-WL. Supervised, H-WL. Helped design figures, MN and H-WL. Edited, H-WL. All authors have read and agreed to the published version of the manuscript.

## Funding

The authors acknowledge funding support by NIH grants 1T32CA247819 (KW, AR), R01CA228137-01A1 (H-WL), and P30CA012197 (BP), as well as, DoD grants, W81XWH-17-1-0044 (H-WL), W81XWH-19-1-0072 (H-WL), W81XWH-19-1-0753 (H-WL), and W81XWH-20-1-0044 (H-WL).

## Acknowledgments

The authors would like to thank the Carpenter Library at Wake Forest University School of Medicine for open-access literature support.

## Conflict of interest

The authors declare that the research was conducted in the absence of any commercial or financial relationships that could be construed as a potential conflict of interest.

## Publisher’s note

All claims expressed in this article are solely those of the authors and do not necessarily represent those of their affiliated organizations, or those of the publisher, the editors and the reviewers. Any product that may be evaluated in this article, or claim that may be made by its manufacturer, is not guaranteed or endorsed by the publisher.
